# Desempenho do Escore MAGGIC em Indivíduos com Insuficiência Cardíaca: Validação em uma População Brasileira

**DOI:** 10.36660/abc.20250614

**Published:** 2026-04-01

**Authors:** Gabriela Gomes de Paula, Henrique Iahnke Garbin, Maria Eduarda Kaminski, Dayana Dias Mendonça, Gabriela Corrêa Souza, Luis Eduardo Rohde, Luis Beck da Silva, Andreia Biolo

**Affiliations:** 1 Faculdade de Medicina Universidade Federal do Rio Grande do Sul Porto Alegre RS Brasil Faculdade de Medicina, Universidade Federal do Rio Grande do Sul (UFRGS), Porto Alegre, RS – Brasil; 2 Programa de Pós-Graduação em Cardiologia e Ciências Cardiovasculares Universidade Federal do Rio Grande do Sul Porto Alegre Brasil Programa de Pós-Graduação em Cardiologia e Ciências Cardiovasculares, Universidade Federal do Rio Grande do Sul (UFRGS), Porto Alegre, RS – Brasil; 3 Divisão Cardiovascular Hospital de Clínicas de Porto Alegre Porto Alegre RS Brasil Divisão Cardiovascular, Hospital de Clínicas de Porto Alegre, Porto Alegre, RS – Brasil

**Keywords:** Insuficiência Cardíaca, Prognóstico, Medição de Risco, Estudo de Validação

## Abstract

**Fundamento:**

O escore *Meta-Analysis Global Group in Chronic Heart Failure* (MAGGIC) é uma ferramenta de estratificação de risco utilizada para prever mortalidade na insuficiência cardíaca (IC). Entretanto, possíveis diferenças relacionadas ao sexo em seu desempenho e sua aplicabilidade à população brasileira permanecem incertas.

**Objetivos:**

Avaliar diferenças baseadas no sexo no desempenho do escore MAGGIC e validar o escore em uma coorte brasileira de IC.

**Métodos:**

Este estudo de coorte retrospectivo incluiu 866 pacientes acompanhados em um ambulatório de IC. O desfecho primário foi mortalidade por todas as causas em 3 anos. O escore MAGGIC foi calculado para cada paciente. A discriminação foi avaliada por meio da área sob a curva característica de operação do receptor, e a calibração foi avaliada pelo teste de Hosmer-Lemeshow. As análises foram realizadas para a coorte total e estratificadas por sexo. Considerou-se valor de p < 0,05 como estatisticamente significativo.

**Resultados:**

A taxa global de mortalidade em 3 anos foi de 33,4% (36,4% em homens e 27,8% em mulheres; p = 0,010). A mortalidade predita foi de 20,9% (escore médio de 18,3 ± 7), sendo 22,7% em homens e 19,1% em mulheres. O escore demonstrou boa discriminação (área sob a curva = 0,72; intervalo de confiança de 95%: 0,686-0,754), com desempenho semelhante em homens (0,704 [0,661-0,747]) e mulheres (0,733 [0,674-0,792]). A calibração mostrou boa concordância: qui-quadrado (χ^2^) global = 1,1 (p = 0,998), χ^2^ para homens = 0,9 (p = 0,999) e χ^2^ para mulheres = 1,3 (p = 0,995). A mortalidade observada foi maior nos grupos de risco moderado, sem diferença significativa entre os grupos de risco moderado e alto (p = 0,236).

**Conclusão:**

O escore MAGGIC apresentou bom desempenho em uma coorte brasileira de IC, sem diferenças significativas relacionadas ao sexo, embora tenha sido identificada maior mortalidade observada entre pacientes de risco moderado.

## Graphical abstract

**Figura Central:**
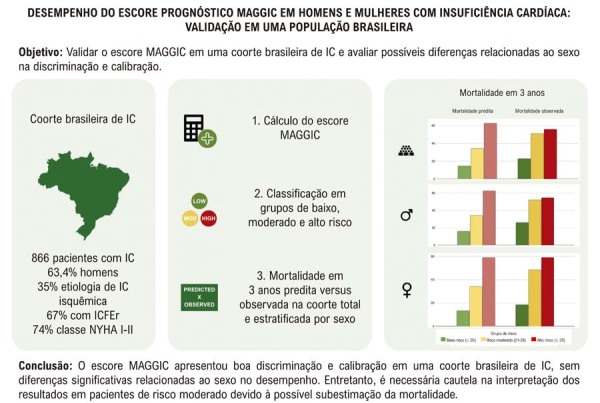
Desempenho do Escore MAGGIC em Indivíduos com Insuficiência Cardíaca: Validação em uma População Brasileira

Desempenho do Escore MAGGIC em Indivíduos com Insuficiência Cardíaca: Validação em uma População Brasileira. IC: insuficiência cardíaca; ICFEr: IC com fração de ejeção reduzida; MAGGIC: Meta-Analysis Global Group in Chronic Heart Failure; NYHA: New York Heart Association.

## Introdução

A insuficiência cardíaca (IC) é uma síndrome clínica que reduz substancialmente a qualidade de vida e está associada a alta utilização de serviços de saúde e custos consideráveis. Embora a incidência de IC tenha se estabilizado ou diminuído ao longo do tempo, sua prevalência continua a aumentar, e a morbidade e a mortalidade permanecem elevadas.^1 - 2^ A avaliação prognóstica é essencial para orientar a tomada de decisão clínica, incluindo estratégias terapêuticas e discussões sobre intervenções avançadas ou cuidados paliativos.^3 - 5^ Para apoiar esse processo, diversos escores prognósticos de risco para IC foram desenvolvidos.^6^ Entre eles, o escore *Meta-Analysis Global Group in Chronic Heart Failure* (MAGGIC) é uma das ferramentas mais robustas e amplamente validadas, utilizadas globalmente.

O escore MAGGIC foi derivado de uma análise combinada de 39.372 pacientes com IC provenientes de 30 estudos de coorte. O MAGGIC incorpora 13 preditores de mortalidade e disponibiliza uma ferramenta online para estimar a mortalidade por todas as causas em 1 e 3 anos.^7^ Embora o escore MAGGIC tenha passado por ampla validação internacional,^8 - 23^ sua aplicação no Brasil ainda é limitada. Até o momento, apenas um estudo avaliou seu desempenho em uma coorte brasileira, com foco em pacientes com fração de ejeção do ventrículo esquerdo (FEVE) < 50%.^24^ Esse estudo analisou mortalidade cardiovascular e hospitalização por IC, e não mortalidade por todas as causas, incluiu um período de seguimento mais curto e apresentou tamanho amostral relativamente pequeno. Essas limitações ressaltam a necessidade de estudos maiores e com poder estatístico adequado para determinar a aplicabilidade do escore à população brasileira mais ampla com IC.

O sexo é um determinante bem estabelecido na IC, influenciando epidemiologia, fatores de risco, fisiopatologia, resposta terapêutica e prognóstico.^25^ Entretanto, permanece incerto se escores prognósticos de risco em IC apresentam desempenho diferente entre homens e mulheres. Poucos estudos investigaram especificamente diferenças relacionadas ao sexo na acurácia preditiva de modelos prognósticos em IC.^16^ Portanto, este estudo teve como objetivo avaliar o desempenho do escore prognóstico MAGGIC em homens e mulheres com IC crônica em acompanhamento ambulatorial em um hospital brasileiro.

## Métodos

### Desenho do estudo e população

Este estudo de coorte incluiu pacientes consecutivos com idade ≥ 18 anos e diagnóstico de IC, independentemente da fração de ejeção, acompanhados em um ambulatório de IC do Hospital de Clínicas de Porto Alegre (HCPA), hospital universitário terciário localizado na cidade de Porto Alegre, Brasil. Os pacientes foram incluídos entre 2008 e 2014.

O desfecho primário foi mortalidade por todas as causas em 3 anos.

### Coleta de dados

Os dados foram extraídos de prontuários eletrônicos e incluíram classe funcional da *New York Heart Association* (NYHA), etiologia da IC, uso de medicamentos, comorbidades, achados laboratoriais, parâmetros ecocardiográficos e informações sociodemográficas. Dados de seguimento foram obtidos a partir dos prontuários eletrônicos ou, para pacientes sem acompanhamento regular no ambulatório, do Sistema de Informações sobre Mortalidade da Secretaria de Estado de Saúde do Rio Grande do Sul, que fornece informações sobre a data e a causa básica do óbito para todos os óbitos registrados no estado.

O estudo foi conduzido de acordo com a Declaração de Helsinque de 2013. O protocolo foi aprovado pelo Comitê de Ética em Pesquisa com Seres Humanos do HCPA (CAAE: 42184921.4.0000.5327). Todos os pacientes forneceram consentimento informado por escrito.

### Análise estatística

Estatísticas descritivas foram utilizadas para resumir as características clínicas e sociodemográficas da coorte total e estratificadas por sexo. Variáveis contínuas foram expressas como média ± desvio padrão ou mediana e intervalo interquartil, enquanto variáveis categóricas foram apresentadas como frequências absolutas e porcentagens. A normalidade foi avaliada pelo teste de Shapiro-Wilk. Comparações entre homens e mulheres foram realizadas por meio do teste *t* de Student não pareado para variáveis contínuas e do teste do qui-quadrado (χ^2^) para variáveis categóricas. Para variáveis com distribuição não normal, foi utilizado o teste de Mann-Whitney *U* .

O escore MAGGIC foi calculado para cada paciente de acordo com o modelo original, utilizando 13 variáveis: idade, fração de ejeção, classe NYHA, creatinina, diabetes, pressão arterial sistólica, índice de massa corporal, duração da IC, tabagismo atual, doença pulmonar obstrutiva crônica, sexo masculino, não uso de betabloqueador e não uso de inibidores da enzima conversora de angiotensina (IECA) ou bloqueadores do receptor de angiotensina (BRA). Para avaliar o desempenho preditivo, os pacientes foram divididos em tercis e categorizados em três grupos de risco: baixo (escore ≤ 20), moderado (21-28) e alto (≥ 29). A mortalidade predita para cada grupo de risco foi obtida a partir do estudo original com base no escore inteiro médio e comparada com a mortalidade observada.

A calibração foi avaliada por meio do teste de qualidade de ajuste de Hosmer-Lemeshow, que agrupou os pacientes em dez decis e avaliou a concordância entre mortalidade observada e predita em cada decil. A discriminação foi avaliada por meio da análise da curva característica de operação do receptor (ROC). A área sob a curva (AUC) com ICs de 95% foi calculada.

A análise de sobrevida foi realizada pelo método de Kaplan-Meier, e as curvas de sobrevida foram comparadas pelo teste de *log-rank* . As curvas de Kaplan-Meier foram estratificadas de acordo com os três grupos de risco predefinidos para mortalidade em 3 anos.

Todas as análises foram realizadas para a coorte total e estratificadas por sexo. Um valor de p bicaudal < 0,05 foi considerado estatisticamente significativo. As análises foram realizadas utilizando o SPSS Statistics for Windows, versão 18.0 (SPSS Inc., Chicago, Ill., EUA).

## Resultados

### Características basais

Um total de 866 pacientes foi incluído, dos quais 63,4% eram homens. As características basais da coorte total e estratificadas por sexo são apresentadas na Tabela 1 . Em comparação com as mulheres, os homens eram significativamente mais velhos. A etiologia da IC diferiu entre os sexos, com maior proporção de IC isquêmica em homens (38,6% vs. 29%) e IC idiopática em mulheres (21% vs. 12%); etiologias hipertensiva e outras também apresentaram variação relacionada ao sexo (23,1% vs. 19,6% e 26,2% vs. 30%, respectivamente). Os homens eram mais clinicamente estáveis (classe NYHA I-II) do que as mulheres, mas apresentavam menor FEVE média, e maior proporção de homens tinha IC com FEVE reduzida (< 40%). Em relação às comorbidades, os homens apresentaram maior prevalência de doença renal crônica (DRC) e fibrilação atrial. Diversos parâmetros laboratoriais diferiram significativamente entre os sexos. Homens receberam com menor frequência prescrição de IECA ou BRA e espironolactona em comparação às mulheres.

**Tabela 1:** – Características basais da população do estudo segundo o sexo

Variable	Total (n = 866)	Homens (n = 549)	Mulheres (n = 317)	Valor p
**Idade, anos**	60,2 ± 13,5	61 ± 12,8	58,8 ± 14,6	0,026
**Raça branca**	659 (76)	423 (77)	236 (74,4)	0,181
**Tabagista atual**	122 (14)	84 (15,3)	38 (12)	0,212
**Etiologia da IC**				< 0,001
Isquêmica	304 (35)	212 (38,6)	92 (29)	
Hipertensiva	189 (22)	127 (23,1)	62 (19,6)	
Idiopática	134 (15)	66 (12)	68 (21,5)	
Outras	239 (28)	144 (26,2)	95 (30)	
**Classe NYHA**				0,002
I-II	683 (74)	424 (77,2)	214 (67,5)	
III-IV	228 (26)	125 (22,8)	103 (32,5)	
**FEVE, %**	34,6 ± 12,2	32,7 ± 12	37,9 ± 14	< 0,001
**ICFEr**	582 (67)	400 (72,9)	182 (57,4)	< 0,001
**FC, bpm**	72 ± 13,7	73,6 ± 14	74,3 ± 13,2	0,489
**PAS, mmHg**	125 ± 24	125 ± 24	125,8 ± 24	0,674
**PAD, mmHg**	77 ± 14	77,2 ± 14,6	77,7 ± 14,3	0,640
**Duração da IC, meses**	27 (5-58)	28 (5-57)	26 (7-59)	0,792
**Comorbidades**				
HAS	528 (61)	334 (60,8)	194 (61,2)	0,974
Diabetes	302 (35)	185 (33,7)	117 (36,9)	0,378
Dislipidemia	253 (29)	165 (30,1)	88 (27,8)	0,524
DPOC	66 (8)	45 (8,2)	21 (6,6)	0,480
DRC	139 (16)	112 (20,4)	27 (8,5)	< 0,001
IAM prévio	216 (25)	145 (26,4)	71 (22,4)	0,217
FA	236 (27)	184 (33,5)	52 (16,4)	< 0,001
**Dados laboratoriais**				
Creatinina, mg/dl	1,10 (0,90-1,39)	1,42 (0,99-1,50)	1,09 (0,77-1,19)	< 0,001
Sódio, mg/dl	140 ± 3,4	140,3 ± 3,2	140,6 ± 3,5	0,258
Potássio, mg/dl	4,5 ± 0,5	4,6 ± 0,5	4,5 ± 0,5	0,015
Ureia, mg/dl	51 (40-70)	52 (41-72)	49 (37-70,5)	0,037
Colesterol total	179 ± 47	174,9 ± 48,2	186,4 ± 45,3	0,006
HDL-C, mg/dl	42 ± 13	40,6 ± 12,9	45,4 ± 12,2	< 0,001
LDL-C, mg/dl	103 ± 38	100,9 ± 37,7	107,8 ± 38,9	0,046
Triglicerídeos, mg/dl	139 (99-206)	131,5 (97-204)	153,5 (106-214)	0,023
**Medicamentos**				
IECA ou BRA	798 (92)	495 (90,2)	303 (95,6)	0,006
Betabloqueadores	761 (88)	489 (89,1)	272 (85,8)	0,190
Espironolactona	364 (42)	216 (39,3)	148 (46,7)	0,042
Diuréticos	767 (89)	487 (88,7)	280 (88,3)	0,954

Os dados são apresentados como média ± desvio padrão, mediana (intervalo interquartil) ou n (%). DRC: doença renal crônica; DPOC: doença pulmonar obstrutiva crônica; FA: fibrilação atrial; FC: frequência cardíaca; FEVE: fração de ejeção do ventrículo esquerdo; HAS: hipertensão arterial sistêmica; HDL-C: colesterol de lipoproteína de alta densidade; IAM: infarto agudo do miocárdio; IC: insuficiência cardíaca; ICFEr: insuficiência cardíaca com fração de ejeção reduzida; IECA: inibidor da enzima conversora de angiotensina; BRA: bloqueador do receptor de angiotensina; LDL-C: colesterol de lipoproteína de baixa densidade; NYHA: New York Heart Association; PAD: pressão arterial diastólica; PAS: pressão arterial sistólica.

### Mortalidade e estratificação de risco

**Figura 1:**
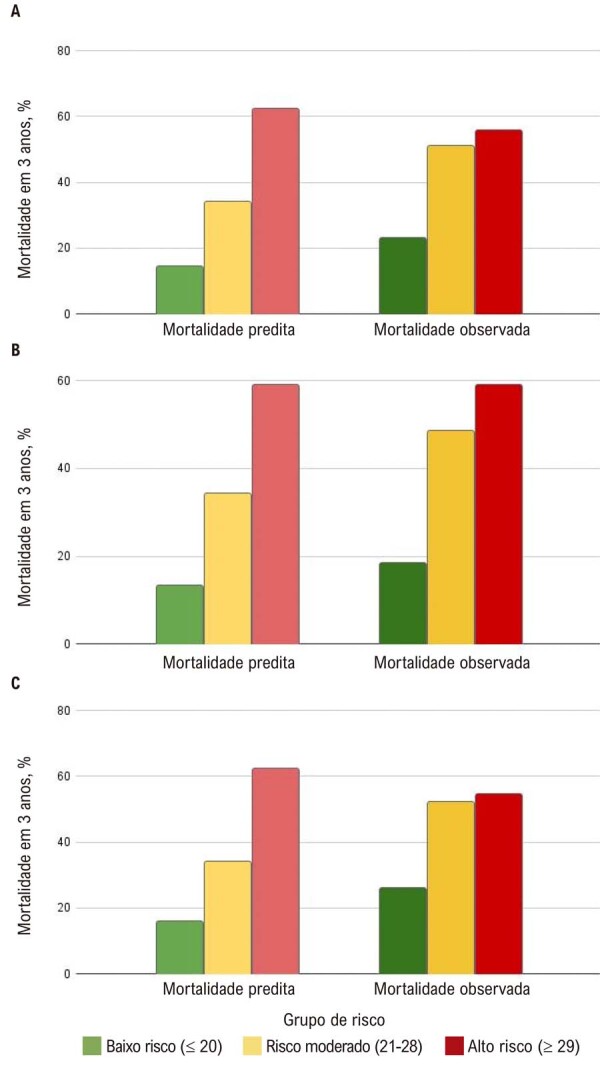
– Comparação entre mortalidade predita e mortalidade observada na coorte total estratificada por sexo. A) Coorte total; B) homens; C) mulheres.

A taxa global de mortalidade em 3 anos foi de 33,4% (36,4% em homens e 27,8% em mulheres [p = 0,01]). A mortalidade predita foi de 20,9% (escore MAGGIC médio de 18,3 ± 7), correspondendo a 22,7% em homens (escore MAGGIC de 19,1 ± 7) e 19,1% em mulheres (escore MAGGIC de 16,8 ± 7). Os pacientes foram categorizados em três grupos de risco, e a mortalidade predita foi comparada com a mortalidade observada ( Figura 1 ). A mortalidade observada foi de 23%, 51,1% e 55,8% nos grupos de baixo, moderado e alto risco, respectivamente (p < 0,001), em comparação com mortalidade predita de 14,6%, 34,2% e 62,5%.

### Discriminação e calibração

A análise da curva ROC demonstrou discriminação adequada, com AUC de 0,72 (IC 95%: 0,686-0,754), com desempenho semelhante em homens (0,704 [0,661-0,747]) e mulheres (0,733 [0,674-0,792]). O escore subestimou a mortalidade nos grupos de baixo e moderado risco e superestimou levemente a mortalidade no grupo de alto risco (Figura 1A). Entre os homens, a mortalidade observada versus predita em 3 anos foi de 26,1% vs. 16%, 52,2% vs. 34,2% e 54,5% vs. 62,5% nos grupos de baixo, moderado e alto risco, respectivamente. Assim como na coorte total, a mortalidade foi subestimada nos grupos de baixo e moderado risco e superestimada no grupo de alto risco, com mortalidade observada semelhante entre as categorias de risco moderado e alto (Figura 1B).

Nas mulheres, o desempenho apresentou padrão ligeiramente diferente: a mortalidade observada versus predita foi de 18,5% vs. 13,4%, 48,5% vs. 34,2% e 59% vs. 59% nos grupos de baixo, moderado e alto risco. Assim como na coorte total e entre os homens, a mortalidade foi subestimada nos grupos de baixo e moderado risco; entretanto, no grupo de alto risco, a mortalidade predita coincidiu com a mortalidade observada (Figura 1C). O teste de Hosmer-Lemeshow indicou boa calibração para a coorte total (χ^2^ = 1,1, p = 0,997), bem como para homens (χ^2^ = 0,9, p = 0,998) e mulheres (χ^2^ = 1,3, p = 0,995).

### Análise de sobrevida

**Figura 2:**
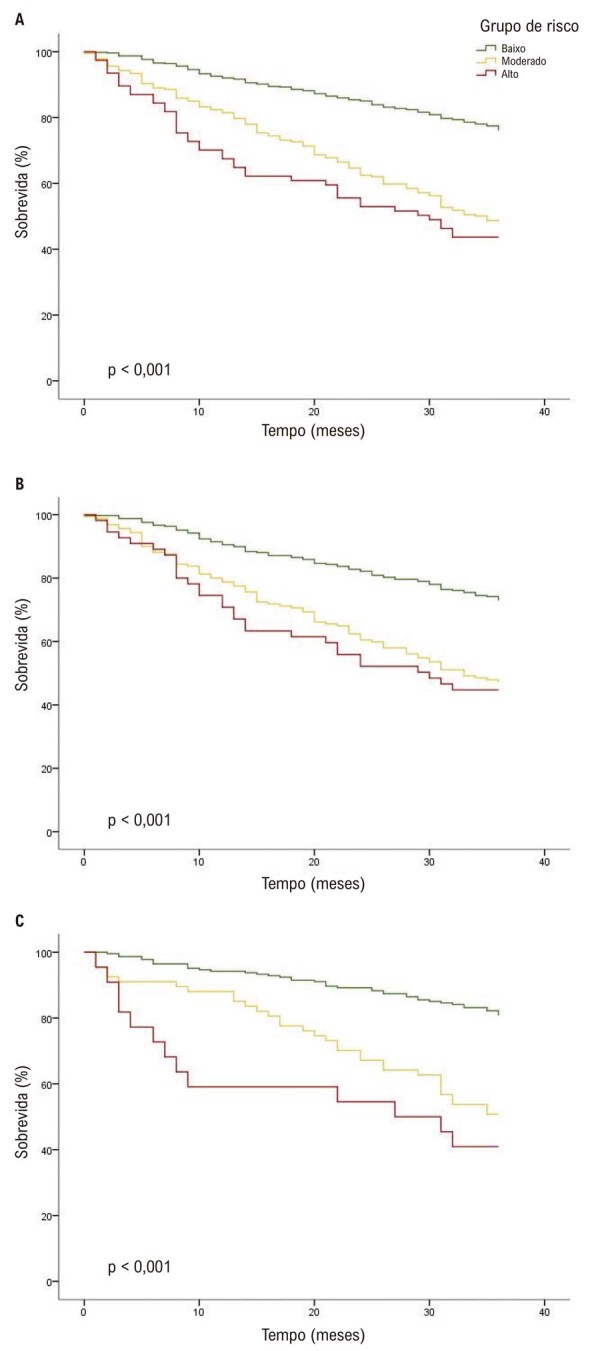
– Curvas de sobrevida de Kaplan-Meier estratificadas pelas categorias de risco do escore Meta-Analysis Global Group in Chronic Heart Failure e por sexo. A) Coorte total; B) homens; C) mulheres.

As curvas de sobrevida de Kaplan-Meier estratificadas pelos três grupos de risco são apresentadas na Figura 2 . Em todas as análises, as taxas de eventos aumentaram significativamente com maior risco predito, com testes de *log-rank* indicando diferenças significativas entre os grupos (p < 0,001). Entretanto, as diferenças entre os grupos de risco moderado e alto não foram estatisticamente significativas (p = 0,24) (Figura Suplementar 1).

## Discussão

Neste estudo, avaliamos o desempenho do escore MAGGIC na predição de mortalidade por todas as causas em 3 anos entre homens e mulheres com IC crônica em uma coorte brasileira. O escore demonstrou boa discriminação e calibração em ambos os sexos, sem diferença significativa no desempenho preditivo entre homens e mulheres. A AUC foi consistente com valores relatados em estudos prévios de validação internacional. Entretanto, a mortalidade observada foi maior no grupo de risco moderado, possivelmente refletindo a maior complexidade clínica dos pacientes acompanhados em um ambulatório especializado em IC em comparação com as populações mais amplas incluídas no desenvolvimento original do escore. Portanto, é necessária cautela na interpretação da categoria de risco intermediário.^21 , 26 , 27^

Estudos prévios de validação relataram valores de índice de concordância e AUC variando entre 0,73 e 0,74 em diferentes populações,^8 , 10 , 11^ incluindo pacientes com IC com fração de ejeção preservada (ICFEp).^12^ Um estudo japonês com pacientes com IC aguda avaliou o escore MAGGIC e uma versão modificada que incorporou peptídeos natriuréticos na alta hospitalar, relatando índice de concordância de 0,71 antes da adição dos biomarcadores.^13^ Outro estudo encontrou índice de concordância de 0,70 em pacientes afro-americanos e 0,72 em pacientes brancos.^14^ Uma análise comparativa entre pacientes na alta hospitalar e ambulatoriais relatou valores de índice de concordância de 0,67 e 0,78, respectivamente.^15^ Um estudo de 2024 comparando múltiplos escores de risco para IC relatou AUC de 0,71 para o escore MAGGIC.^22^ Por outro lado, alguns estudos demonstraram desempenho discriminativo superior. Codina et al.^17^ relataram índice de concordância de 0,80 para mortalidade em 1 ano utilizando o escore MAGGIC. Uma coorte chinesa que avaliou mortalidade em 1 ano relatou AUC de 0,84.^19^ Outro estudo que avaliou predição de mortalidade em 1 ano e readmissão de curto prazo relatou AUC de 0,82 para mortalidade em 1 ano utilizando o escore MAGGIC.^20^

Quando comparado a outros modelos prognósticos amplamente utilizados em IC, o escore MAGGIC apresenta desempenho discriminativo semelhante ao *Seattle Heart Failure Model* , que relatou AUC de 0,73,^29^ e ao estudo PREDICT-HF, que demonstrou valores de AUC de 0,71 em 1 ano e 0,70 em 2 anos.^30^ Entretanto, esses modelos exigem um conjunto mais amplo de variáveis, incluindo parâmetros laboratoriais como hemoglobina, contagem de linfócitos, ácido úrico, colesterol total e peptídeo natriurético tipo B N-terminal, além de informações sobre terapia com dispositivos. Em contraste, o escore MAGGIC baseia-se em variáveis clínicas e laboratoriais prontamente disponíveis, sem necessidade de biomarcadores extensos ou dados de dispositivos, tornando-o mais prático e facilmente aplicável, especialmente em cenários com recursos limitados. Isso facilita a estratificação de risco, particularmente na distinção entre pacientes de baixo risco e aqueles de risco moderado ou alto que podem se beneficiar de manejo mais precoce e intensivo.

Apenas uma validação prévia do escore MAGGIC no Brasil foi relatada, sendo limitada por pequeno tamanho amostral (n = 93), inclusão exclusiva de pacientes com IC com fração de ejeção reduzida (ICFEr) e definição de desfecho diferente da utilizada no modelo original.^24^ Em vez de avaliar mortalidade por todas as causas em 1 e 3 anos, aquele estudo analisou mortalidade ou hospitalização por IC em 6 meses, resultando em AUC inferior (0,59). Em contraste, nosso estudo incluiu uma coorte maior de quase 900 pacientes, abrangendo tanto ICFEr quanto ICFEp, avaliou mortalidade por todas as causas em 3 anos e demonstrou melhor desempenho discriminativo. O escore MAGGIC mostrou-se útil para identificar pacientes de baixo risco nesta coorte brasileira. Contudo, o modelo apresentou limitações na estratificação precisa de indivíduos de risco moderado, pois a mortalidade nesse grupo foi subestimada. Embora o desempenho tenha sido robusto para identificar pacientes de baixo risco, a predição na categoria de risco moderado pode ser menos confiável.

Nosso estudo examinou especificamente o desempenho do escore MAGGIC segundo o sexo, uma área ainda pouco explorada.^16^ Não observamos diferenças estatisticamente significativas na discriminação ou calibração entre homens e mulheres, em concordância com achados prévios. É importante destacar que nossa coorte incluiu pacientes independentemente da FEVE, enquanto validação prévia baseada em sexo foi conduzida em uma coorte canadense restrita à ICFEr. Observou-se que homens apresentaram menor uso de IECA ou BRA e espironolactona em comparação às mulheres. Essa diferença pode ser explicada pela maior prevalência de DRC e níveis mais elevados de potássio sérico entre os homens, o que pode ter limitado o uso dessas terapias devido a preocupações com segurança e tolerabilidade.

Algumas limitações devem ser reconhecidas. Tanto o escore MAGGIC original quanto nossa coorte foram derivados antes da ampla adoção de sacubitril-valsartana e inibidores de SGLT2. O tratamento da IC evoluiu substancialmente desde a coleta dos dados basais, e a avaliação em coortes mais contemporâneas pode fornecer informações adicionais. Além disso, este foi um estudo de centro único, sendo necessária validação multicêntrica para confirmar a generalização dos achados no Brasil, país caracterizado por significativa diversidade regional.

## Conclusão

Nossos achados indicam que o escore MAGGIC é uma ferramenta válida para predizer mortalidade em 3 anos em pacientes brasileiros com IC e apresenta boa discriminação e calibração em homens e mulheres. O desempenho foi consistente com validações internacionais prévias, e não foram identificadas diferenças significativas relacionadas ao sexo. Esses resultados apoiam o uso do escore MAGGIC na população brasileira com IC; entretanto, é necessária cautela na interpretação dos resultados em pacientes de risco moderado devido à possível subestimação da mortalidade. Validação multicêntrica é necessária para confirmar sua aplicabilidade em diferentes regiões do Brasil.

## Material suplementar

Material suplementar
