# Supplementary material for: Desempenho do Escore MAGGIC em Indivíduos com Insuficiência Cardíaca: Validação em uma População Brasileira
Source: Arq Bras Cardiol. 2026 Apr 1;123(3):e20250614. [Article in Portuguese] doi: 10.36660/abc.20250614 (PMC13128214; doi:10.36660/abc.20250614)
Supplement: Material suplementar [file 0066-782x-abc-123-3-e20250614-suppl01.pdf]

Supplemental material

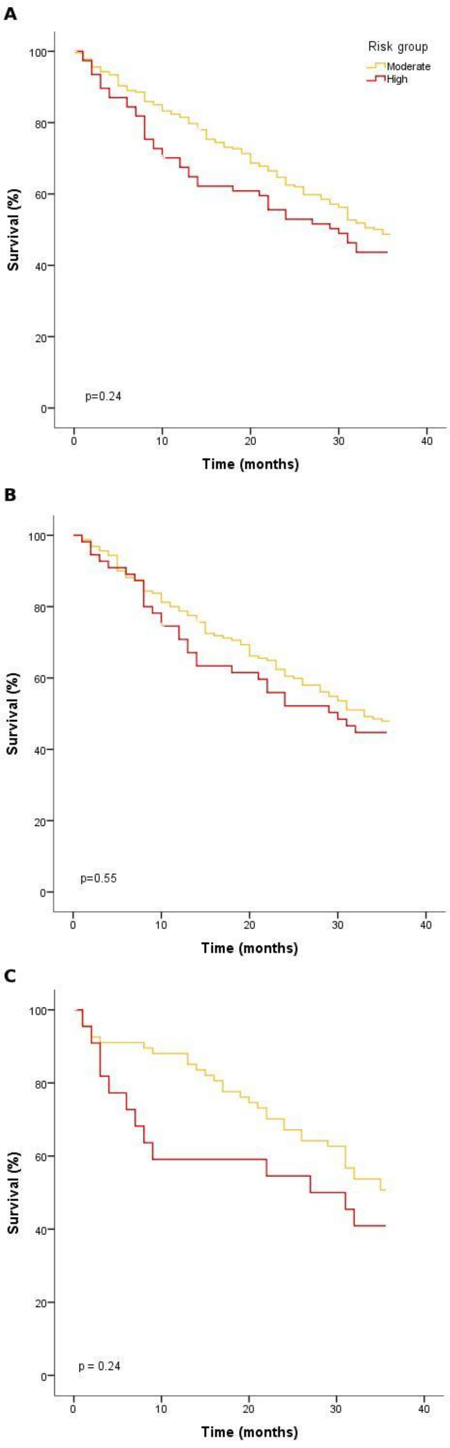

Supplemental material. Kaplan-Meier Survival Curves Stratified by MAGGIC moderate- and high-risk groups and Sex. 2A. Overall cohort. 2B. Male patients. 2C. Female patients.
