# Supplementary material for: Desempenho do Escore MAGGIC em Indivíduos com Insuficiência Cardíaca: Validação em uma População Brasileira
Source: Arq Bras Cardiol. 2026 Apr 1;123(3):e20250614. [Article in Portuguese] doi: 10.36660/abc.20250614 (PMC13128214; doi:10.36660/abc.20250614)
Supplement: Translation

# Translation: Performance of the MAGGIC Score in Individuals with Heart Failure: Validation in a Brazilian Population

**DOI:** 10.36660/abc.20250614i

**Authors:** Gabriela Gomes de Paula, Henrique Iahnke Garbin, Maria Eduarda Kaminski, Dayana Dias Mendonça, Gabriela Corrêa Souza, Luis Eduardo Rohde, Luis Beck da Silva, Andreia Biolo

**Affiliations:** 1 Brazil Faculdade de Medicina, Universidade Federal do Rio Grande do Sul (UFRGS), Porto Alegre, RS – Brazil; 2 Brazil Programa de Pós-Graduação em Cardiologia e Ciências Cardiovasculares, Universidade Federal do Rio Grande do Sul (UFRGS), Porto Alegre, RS – Brazil; 3 Brazil Divisão Cardiovascular, Hospital de Clínicas de Porto Alegre, Porto Alegre, RS – Brazil

**Keywords:** Heart Failure, Prognosis, Risk Assessment, Validation Study

## Abstract

**Background:**

The Meta-Analysis Global Group in Chronic Heart Failure (MAGGIC) score is a risk stratification tool used to predict mortality in heart failure (HF). However, potential sex-related differences in its performance and its applicability to the Brazilian population remain uncertain.

**Objectives:**

To evaluate sex-based differences in the performance of the MAGGIC score and to validate the score in a Brazilian HF cohort.

**Methods:**

This retrospective cohort study included 866 patients followed at a HF outpatient clinic. The primary outcome was 3-year all-cause mortality. The MAGGIC score was calculated for each patient. Discrimination was assessed using the area under the receiver operating characteristic curve, and calibration was evaluated using the Hosmer-Lemeshow test. Analyses were performed for the overall cohort and stratified by sex. A p-value < 0.05 was considered statistically significant.

**Results:**

The overall 3-year mortality rate was 33.4% (36.4% in men and 27.8% in women; p = 0.010). Predicted mortality was 20.9% (mean score 18.3 ± 7), with 22.7% for men and 19.1% for women. The score demonstrated good discrimination (area under the curve = 0.72; 95% CI: 0.686-0.754), with similar performance in men (0.704 [0.661-0.747]) and women (0.733 [0.674-0.792]). Calibration showed good agreement: overall chi-square (χ^2^) = 1.1 (p = 0.998), men χ^2^= 0.9 (p = 0.999), and women χ^2^= 1.3 (p = 0.995). Observed mortality was higher in moderate-risk groups, with no significant difference between moderate- and high-risk groups (p = 0.236).

**Conclusion:**

The MAGGIC score showed good performance in a Brazilian HF cohort, with no significant sex-based differences, although higher observed mortality was identified among moderate-risk patients.

## Introduction

Heart failure (HF) is a clinical syndrome that substantially reduces quality of life and is associated with high health care utilization and considerable costs. Although the incidence of HF has stabilized or declined over time, its prevalence continues to rise, and morbidity and mortality remain high.^1 - 2^Prognostic assessment is essential for guiding clinical decision-making, including therapeutic strategies and discussions regarding advanced interventions or palliative care.^3 - 5^To support this process, several prognostic risk scores for HF have been developed.^6^Among these, the Meta-Analysis Global Group in Chronic Heart Failure (MAGGIC) score is one of the most robust, widely validated tools used globally.

The MAGGIC score was derived from a pooled analysis of 39,372 patients with HF across 30 cohort studies. MAGGIC incorporates 13 predictors of mortality and provides an online tool to estimate all-cause mortality at 1 and 3 years.^7^Although the MAGGIC score has undergone extensive international validation,^8 - 23^its application in Brazil remains limited. To date, only one study has evaluated its performance in a Brazilian cohort, focusing on patients with left ventricular ejection fraction (LVEF) < 50%.^24^That study examined cardiovascular mortality and HF hospitalization rather than all-cause mortality, included a shorter follow-up period, and had a relatively small sample size. These limitations underscore the need for larger, well-powered studies to determine the score’s applicability to the broader Brazilian HF population.

Sex is a well-established determinant in HF, influencing epidemiology, risk factors, pathophysiology, therapeutic response, and prognosis.^25^However, whether prognostic HF risk scores perform differently in men and women remains uncertain. Few studies have specifically investigated sex-related differences in the predictive accuracy of HF prognostic models.^16^Therefore, this study aimed to evaluate the performance of the MAGGIC prognostic score in men and women with chronic HF receiving outpatient care at a Brazilian hospital.

## Methods

### Study design and population

This cohort study included consecutive patients aged ≥ 18 years with a diagnosis of HF, regardless of ejection fraction, followed at a HF outpatient clinic at Hospital de Clínicas de Porto Alegre (HCPA), a tertiary university hospital in the city of Porto Alegre, Brazil. Patients were enrolled between 2008 and 2014.

The primary outcome was 3-year all-cause mortality.

### Data collection

Data were extracted from electronic medical records and included New York Heart Association (NYHA) functional class, HF etiology, medication use, comorbidities, laboratory findings, echocardiographic parameters, and sociodemographic information. Follow-up data were obtained from electronic medical records or, for patients not under regular follow-up at the clinic, from the Mortality Information System of the Health Department of the State of Rio Grande do Sul, which provides information on the date and primary cause of death for all deaths registered in the state.

The study was conducted in accordance with the 2013 Declaration of Helsinki. The protocol was approved by the Human Research Ethics Committee at HCPA (CAAE: 42184921.4.0000.5327). All patients provided written informed consent.

### Statistical analysis

Descriptive statistics were used to summarize the clinical and sociodemographic characteristics of the overall cohort and stratified by sex. Continuous variables were expressed as mean ± standard deviation or median and interquartile range, while categorical variables were presented as absolute counts and percentages. Normality was assessed using the Shapiro-Wilk test. Comparisons between men and women were performed using the unpaired Student’s *t* test for continuous variables and the chi-square (χ^2^) test for categorical variables. For non-normally distributed variables, the Mann-Whitney *U* test was used.

The MAGGIC score was calculated for each patient according to the original model, using 13 variables: age, ejection fraction, NYHA class, creatinine, diabetes, systolic blood pressure, body mass index, HF duration, current smoking, chronic obstructive pulmonary disease, male sex, non-use of a beta-blocker, and non-use of angiotensin-converting enzyme inhibitors (ACEi) or angiotensin receptor blockers (ARB). To evaluate predictive performance, patients were divided into tertiles and categorized into three risk groups: low (score ≤ 20), moderate (21-28), and high (≥ 29). Predicted mortality for each risk group was obtained from the original study based on the mean integer score and compared with observed mortality.

Calibration was assessed using the Hosmer-Lemeshow goodness-of-fit test, which grouped patients into ten deciles and evaluated agreement between observed and predicted mortality within each decile. Discrimination was evaluated using receiver operating characteristic (ROC) curve analysis. The area under the curve (AUC) with 95% CIs was calculated.

Survival analysis was performed using the Kaplan-Meier method, and survival curves were compared using the log-rank test. Kaplan-Meier curves were stratified according to the three predefined risk groups for 3-year mortality.

All analyses were conducted for the overall cohort and stratified by sex. A two-sided p-value < 0.05 was considered statistically significant. Analyses were performed using SPSS Statistics for Windows, version 18.0 (SPSS Inc., Chicago, Ill., USA).

## Results

### Baseline characteristics

A total of 866 patients were included, of whom 63.4% were men. Baseline characteristics for the overall cohort and stratified by sex are presented in Table 1 . Compared with women, men were significantly older. HF etiology differed between sexes, with higher proportions of ischemic HF in men (38.6% vs. 29%) and idiopathic HF in women (21% vs. 12%); hypertensive and other etiologies also showed sex-related variation (23.1% vs. 19.6% and 26.2% vs. 30%, respectively). Men were more clinically stable (NYHA class I-II) than women but had lower mean LVEF, and a greater proportion of men had HF with reduced LVEF (< 40%). Regarding comorbidities, men had higher prevalence of chronic kidney disease (CKD) and atrial fibrillation. Several laboratory parameters differed significantly between sexes. Men were less frequently prescribed ACEi or ARB and spironolactone than women.

**Table 1:** – Baseline characteristics of the study population according to sex

Variable	Total (n = 866)	Men (n = 549)	Women (n = 317)	p-value
**Age, years**	60.2 ± 13.5	61 ± 12.8	58.8 ± 14.6	0.026
**White race**	659 (76)	423 (77)	236 (74.4)	0.181
**Current smoker**	122 (14)	84 (15.3)	38 (12)	0.212
**HF etiology**				< 0.001
Ischemic	304 (35)	212 (38.6)	92 (29)	
Hypertensive	189 (22)	127 (23.1)	62 (19.6)	
Idiopathic	134 (15)	66 (12)	68 (21.5)	
Others	239 (28)	144 (26.2)	95 (30)	
**NYHA class**				0.002
I-II	683 (74)	424 (77.2)	214 (67.5)	
III-IV	228 (26)	125 (22.8)	103 (32.5)	
**LVEF, %**	34.6 ± 12.2	32.7 ± 12	37.9 ± 14	< 0.001
**HFrEF**	582 (67)	400 (72.9)	182 (57.4)	< 0.001
**HR, bpm**	72 ± 13.7	73.6 ± 14	74.3 ± 13.2	0.489
**SBP, mmHg**	125 ± 24	125 ± 24	125.8 ± 24	0.674
**DBP, mmHg**	77 ± 14	77.2 ± 14.6	77.7 ± 14.3	0.640
**HF duration, months**	27 (5-58)	28 (5-57)	26 (7-59)	0.792
**Comorbidities**				
Hypertension	528 (61)	334 (60.8)	194 (61.2)	0.974
Diabetes	302 (35)	185 (33.7)	117 (36.9)	0.378
Dyslipidemia	253 (29)	165 (30.1)	88 (27.8)	0.524
COPD	66 (8)	45 (8.2)	21 (6.6)	0.480
CKD	139 (16)	112 (20.4)	27 (8.5)	< 0.001
Prior MI	216 (25)	145 (26.4)	71 (22.4)	0.217
AF	236 (27)	184 (33.5)	52 (16.4)	< 0.001
**Laboratory data**				
Creatinine, mg/dL	1.10 (0.90-1.39)	1.42 (0.99-1.50)	1.09 (0.77-1.19)	< 0.001
Sodium, mg/dL	140 ± 3.4	140.3 ± 3.2	140.6 ± 3.5	0.258
Potassium, mg/dL	4.5 ± 0.5	4.6 ± 0.5	4.5 ± 0.5	0.015
Urea, mg/dL	51 (40-70)	52 (41-72)	49 (37-70.5)	0.037
Total cholesterol	179 ± 47	174.9 ± 48.2	186.4 ± 45.3	0.006
HDL-C, mg/dL	42 ± 13	40.6 ± 12.9	45.4 ± 12.2	< 0.001
LDL-C, mg/dL	103 ± 38	100.9 ± 37.7	107.8 ± 38.9	0.046
Triglycerides, mg/dL	139 (99-206)	131.5 (97-204)	153.5 (106-214)	0.023
**Drugs**				
ACEi or ARB	798 (92)	495 (90.2)	303 (95.6)	0.006
Beta-blockers	761 (88)	489 (89.1)	272 (85.8)	0.190
Spironolactone	364 (42)	216 (39.3)	148 (46.7)	0.042
Diuretics	767 (89)	487 (88.7)	280 (88.3)	0.954

Data are presented as mean ± standard deviation, median (interquartile range), or n (%). AF: atrial fibrillation; ACEi: angiotensin-converting enzyme inhibitor; ARB: angiotensin receptor blocker; CKD: chronic kidney disease; COPD: chronic obstructive pulmonary disease; DBP: diastolic blood pressure; HDL-C: high-density lipoprotein cholesterol; HF: heart failure; HFrEF: HF with reduced ejection fraction; HR: heart rate; LDL-C: low-density lipoprotein cholesterol; LVEF: left ventricular ejection fraction; MI: myocardial infarction; NYHA: New York Heart Association; SBP: systolic blood pressure.

### Mortality and risk stratification

The overall 3-year mortality rate was 33.4% (36.4% in men and 27.8% in women [p = 0.01]). Predicted mortality was 20.9% (mean MAGGIC score 18.3 ± 7), corresponding to 22.7% in men (MAGGIC score 19.1 ± 7) and 19.1% in women (MAGGIC score 16.8 ± 7). Patients were categorized into three risk groups, and predicted mortality was compared with observed mortality ( Figure 1 ). Observed mortality was 23%, 51.1%, and 55.8% in the low-, moderate-, and high-risk groups, respectively (p < 0.001), compared with predicted mortality of 14.6%, 34.2%, and 62.5%.

**Figure 1:** – Comparison of predicted versus observed mortality in the overall cohort and stratified by sex. A) Overall cohort; B) men; C) women.

### Discrimination and calibration

ROC curve analysis demonstrated adequate discrimination, with an AUC of 0.72 (95% CI: 0.686-0.754), with similar performance in men (0.704 [0.661-0.747]) and women (0.733 [0.674-0.792]). The score underestimated mortality in the low- and moderate-risk groups and slightly overestimated mortality in the high-risk group (Figure 1A). Among men, observed versus predicted 3-year mortality was 26.1% vs. 16%, 52.2% vs. 34.2%, and 54.5% vs. 62.5% across the low-, moderate-, and high-risk groups, respectively. As in the overall cohort, mortality was underestimated in the low- and moderate-risk groups and overestimated in the high-risk group, with similar observed mortality between moderate- and high-risk categories (Figure 1B).

In women, performance showed a slightly different pattern: observed versus predicted mortality was 18.5% vs. 13.4%, 48.5% vs. 34.2%, and 59% vs. 59% across the low-, moderate-, and high-risk groups. As in the overall cohort and among men, mortality was underestimated in the low- and moderate-risk groups; however, in the high-risk group, predicted mortality matched observed mortality (Figure 1C). The Hosmer-Lemeshow test indicated good calibration for the overall cohort (χ^2^= 1.1, p = 0.997) as well as for men (χ^2^= 0.9, p = 0.998) and women (χ^2^= 1.3, p = 0.995).

### Survival analysis

Kaplan-Meier survival curves stratified by the three risk groups are shown in Figure 2 . Across all analyses, event rates increased significantly with higher predicted risk, with log-rank tests indicating significant differences among groups (p < 0.001). However, differences between the moderate- and high-risk groups were not statistically significant (p = 0.24) (Supplementary Figure 1 ).

**Figure 2:** – Kaplan-Meier survival curves stratified by Meta-Analysis Global Group in Chronic Heart Failure score risk categories and sex. A) Overall cohort; B) men; C) women.

## Discussion

In this study, we evaluated the performance of the MAGGIC score in predicting 3-year all-cause mortality among men and women with chronic HF in a Brazilian cohort. The score demonstrated good discrimination and calibration in both sexes, with no significant difference in predictive performance between men and women. The AUC was consistent with values reported in previous international validation studies. However, observed mortality was higher in the moderate-risk group, possibly reflecting the greater clinical complexity of patients followed at a specialized HF outpatient clinic compared with the broader populations included in the score’s original development. Therefore, caution is warranted when interpreting the intermediate-risk category.^21 , 26 , 27^

Previous validation studies have reported C-index and AUC values ranging from 0.73 to 0.74 across different populations,^8 , 10 , 11^including patients with HF with preserved ejection fraction (HFpEF).^12^A Japanese study of patients acute with HF evaluated the MAGGIC score and a modified version incorporating discharge natriuretic peptides, and reported a C-index of 0.71 before adding biomarkers.^13^Another study found a C-index of 0.70 in African American patients and 0.72 in White patients.^14^A comparative analysis of patients at hospital discharge and outpatients reported C-index values of 0.67 and 0.78, respectively.^15^A 2024 study comparing multiple risk scores for HF reported an AUC of 0.71 for the MAGGIC score.^22^Conversely, some studies have demonstrated higher discriminative performance. Codina et al.^17^reported a C-index of 0.80 for 1-year mortality using the MAGGIC score. A Chinese cohort evaluating 1-year mortality reported an AUC of 0.84.^19^Another study assessing prediction of 1-year mortality and short-term readmission reported an AUC of 0.82 for 1-year mortality using the MAGGIC score.^20^

When compared with other widely used HF prognostic models, the MAGGIC score shows similar discriminatory performance to the Seattle Heart Failure Model, which reported an AUC of 0.73,^29^and the PREDICT-HF trial, which showed AUC values of 0.71 at 1 year and 0.70 at 2 years.^30^However, these models require a broader set of variables, including laboratory parameters such as hemoglobin, lymphocyte count, uric acid, total cholesterol, and N-terminal pro-B-type natriuretic peptide as well as information on device therapy. In contrast, the MAGGIC score relies on readily available clinical and laboratory variables without extensive biomarker or device data, making it more practical and easily applicable, particularly in resource-limited settings. This facilitates effective risk stratification, especially in distinguishing low-risk patients from those at moderate or high risk who may benefit from earlier and more intensive management.

Only one prior validation of the MAGGIC score in Brazil has been reported, and it was limited by a small sample (n = 93), exclusive inclusion of patients with HF with reduced ejection fraction (HFrEF), and an outcome definition different from that used in the original model.^24^Instead of evaluating 1- and 3-year all-cause mortality, that study assessed mortality or HF hospitalization at 6 months, resulting in a lower AUC (0.59). In contrast, our study included a larger cohort of nearly 900 patients, encompassing both HFrEF and HFpEF, evaluated 3-year all-cause mortality, and demonstrated improved discriminative performance. The MAGGIC score proved useful in identifying low-risk patients in this Brazilian cohort. However, the model showed limitations in accurately stratifying moderate-risk individuals, as mortality in this group was underestimated. Although performance was robust for identifying low-risk patients, prediction within the moderate-risk category may be less reliable.

Our study specifically examined sex-based performance of the MAGGIC score, an area that remains underexplored.^16^We found no statistically significant differences in discrimination or calibration between men and women, consistent with prior findings. Importantly, our cohort included patients regardless of LVEF, whereas previous sex-based validation was conducted in a Canadian cohort restricted to HFrEF. Notably, men had lower use of ACEi or ARBs and spironolactone than women. This difference may be explained by the higher prevalence of CKD and increased serum potassium levels among men, which may have limited therapy use due to safety and tolerability concerns.

Several limitations should be acknowledged. Both the original MAGGIC score and our cohort were derived before the widespread adoption of sacubitril-valsartan and SGLT2 inhibitors. The treatment of HF has evolved substantially since the baseline data were collected, and evaluation in more contemporary cohorts may provide additional insights. Furthermore, this was a single-center study, and multicenter validation is necessary to confirm generalizability across Brazil, a country characterized by substantial regional diversity.

## Conclusion

Our findings indicate that the MAGGIC score is a valid tool for predicting 3-year mortality in Brazilian patients with HF and demonstrates good discrimination and calibration in both men and women. Performance was consistent with previous international validations, and no significant sex-based differences were identified. These results support the use of the MAGGIC score in the Brazilian HF population; however, caution is warranted when interpreting results in moderate-risk patients due to possible underestimation of mortality. Multicenter validation is needed to confirm applicability across different regions of Brazil.

## Supplemental Materials

Supplemental material
